# Conjugated β-Cyclodextrin Enhances the Affinity of Folic Acid towards FRα: Molecular Dynamics Study

**DOI:** 10.3390/molecules26175304

**Published:** 2021-08-31

**Authors:** Mohammad G. Al-Thiabat, Amirah Mohd Gazzali, Noratiqah Mohtar, Vikneswaran Murugaiyah, Ezatul Ezleen Kamarulzaman, Beow Keat Yap, Noorsaadah Abd Rahman, Rozana Othman, Habibah A. Wahab

**Affiliations:** 1 School of Pharmaceutical Sciences, Universiti Sains Malaysia, George Town 11800, Malaysia; mohd.althiabat@gmail.com (M.G.A.-T.); amirahmg@usm.my (A.M.G.); noratiqah@usm.my (N.M.); vicky@usm.my (V.M.); ezatulezleen@usm.my (E.E.K.); Beowkeat@usm.my (B.K.Y.); 2 Pharmaceutical Design and Simulation (PHDS) Laboratory, School of Pharmaceutical Sciences, Universiti Sains Malaysia, George Town 11800, Malaysia; 3 Department of Chemistry, Faculty of Science, Universiti Malaya, Kuala Lumpur 50603, Malaysia; noorsaadah@um.edu.my; 4 Center for Natural Products Research and Drug Discovery (CENAR), Department of Pharmaceutical Chemistry, Faculty of Pharmacy, Universiti Malaya, Kuala Lumpur 50603, Malaysia

**Keywords:** targeted drug delivery system, folate receptor alpha, folic acid-conjugated cyclodextrins, molecular docking, molecular dynamics, radius of gyration (Rg), H-bonds, MM-PBSA, MM-PBSA per residue energy decomposition

## Abstract

Drug targeting is a progressive area of research with folate receptor alpha (FRα) receiving significant attention as a biological marker in cancer drug delivery. The binding affinity of folic acid (FA) to the FRα active site provides a basis for recognition of FRα. In this study, FA was conjugated to beta-cyclodextrin (βCD) and subjected to in silico analysis (molecular docking and molecular dynamics (MD) simulation (100 ns)) to investigate the affinity and stability for the conjugated system compared to unconjugated and apo systems (ligand free). Docking studies revealed that the conjugated FA bound into the active site of FRα with a docking score (free binding energy < −15 kcal/mol), with a similar binding pose to that of unconjugated FA. Subsequent analyses from molecular dynamics (MD) simulations, root mean square deviation (RMSD), root mean square fluctuation (RMSF), and radius of gyration (Rg) demonstrated that FA and FA–βCDs created more dynamically stable systems with FRα than the apo-FRα system. All systems reached equilibrium with stable RMSD values ranging from 1.9–2.4 Å and the average residual fluctuation values of the FRα backbone atoms for all residues (except for terminal residues ARG8, THR9, THR214, and LEU215) were less than 2.1 Å with a consistent Rg value of around 16.8 Å throughout the MD simulation time (0–100 ns). The conjugation with βCD improved the stability and decreased the mobility of all the residues (except residues 149–151) compared to FA–FRα and apo-FRα systems. Further analysis of H-bonds, binding free energy (MM-PBSA), and per residue decomposition energy revealed that besides APS81, residues HIS20, TRP102, HIS135, TRP138, TRP140, and TRP171 were shown to have more favourable energy contributions in the holo systems than in the apo-FRα system, and these residues might have a direct role in increasing the stability of holo systems.

## 1. Introduction

Chemotherapy remains as an important option in addition to other anticancer treatments including surgery and radiotherapy [1,2]. Although anticancer drugs are available for cancer chemotherapy, many of them have significant toxicity and adverse effects. One of the most promising approaches to overcome adverse effects of anticancer drugs is utilizing targeted drug delivery system (TDDS) [1,3]. This drug delivery system transports the drug selectively to its site of action within the therapeutic concentration, restricting the drug’s access to healthy cells, thus, minimizing the toxic side effects. A TDDS can rapidly enter tumour cells through receptor-mediated endocytosis. One of the receptor targets for internalization of anticancer TDDS is folate receptor alpha (FRα) [4]. This receptor is highly expressed in many human tumour cells including ovary, kidney, breast, myeloid, brain, and lung cancer cells [5]. Conjugation of a drug delivery system with folic acid (FA) has been used to achieve active targeting [6,7] as FA can be recognized by FRα with high binding constants (Kd ∼ 10^10^ M^−1^) [5]. This has led to the development of various folate-appended drug carriers such as liposomes [8,9,10], dendrimers [11,12,13] and micelles [14,15,16].

Folate-conjugated cyclodextrins (FA-CDs) are another attractive drug carrier that can potentially be useful in the TDDS for anticancer drugs. Cyclodextrins (CDs) are degradation products of starch with α-, β-, and γ-CDs being the most common natural CDs, consisting of six, seven, and eight glucose units, respectively. They possess a remarkable ability to incorporate various guest molecules, via noncovalent interactions, into their hydrophobic cavities [17]. Several FA-CD systems for anticancer drugs have been reported [18,19,20] with a recent development of folic acid–polyethylene glycol–β-cyclodextrin (FA–PEG–β-CD) nanoparticles (NPs) by Fan et al. [21], as a drug-delivery system for doxorubicin for liver cancer therapy. The in vitro drug release results showed that the FA–PEG–β-CD NPs improved doxorubicin’s solubility and could also control the drug release. Furthermore, docetaxel-loaded folic acid-conjugated cyclodextrin (FA-CD) developed by Xu et al. [22] has been shown to be more effective in inducing apoptosis in FR-expressing cells.

Ligand conjugated CD development and investigation rely mostly on trial-and-error in the laboratory by formulation scientists, which is time-consuming and costly [23]. The structure, dynamics, and energetics of cyclodextrin complexes may be investigated using a molecular modelling approach [23]. Yin et al. [24], for example, investigated the potential of a novel drug delivery system consisting folic acid-conjugated CD carriers for the delivery of adamantane (Ada) and doxorubicin (DOX), (FACD–Ada–DOX), predicted in silico by molecular docking. The prediction was validated in an in vitro assay where the cellular uptake of these nanoparticles was eight-fold higher in comparison to conventional systems in FR-positive tumour cells via endocytosis. In another study, docking was used to predict the conformation of a POH/β-CD inclusion complex of which the predicted most stable structure (1:1 molar ratio) was selected for formulation and in vitro and in vivo studies [25].

**Figure 1:**
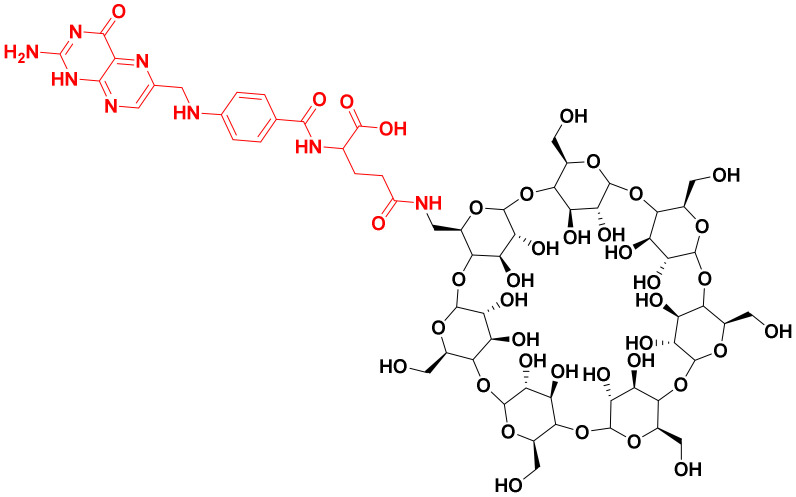
Structure for FA–βCD. Folic acid moiety is in red and β-cyclodextrin is in black.

Hence, in this study, molecular docking and molecular dynamics simulation of conjugated FA to beta-cyclodextrin (βCD) were carried out to understand the effect of conjugation on the stability and interactions of FA with FRα (Figure 1). It is hoped that this study will pave the way for the consideration of folic acid conjugation in order to provide a more selective and targeted drug delivery systems.

## 2. Results and Discussion

### 2.1. Molecular Docking Analysis

**Figure 2:**
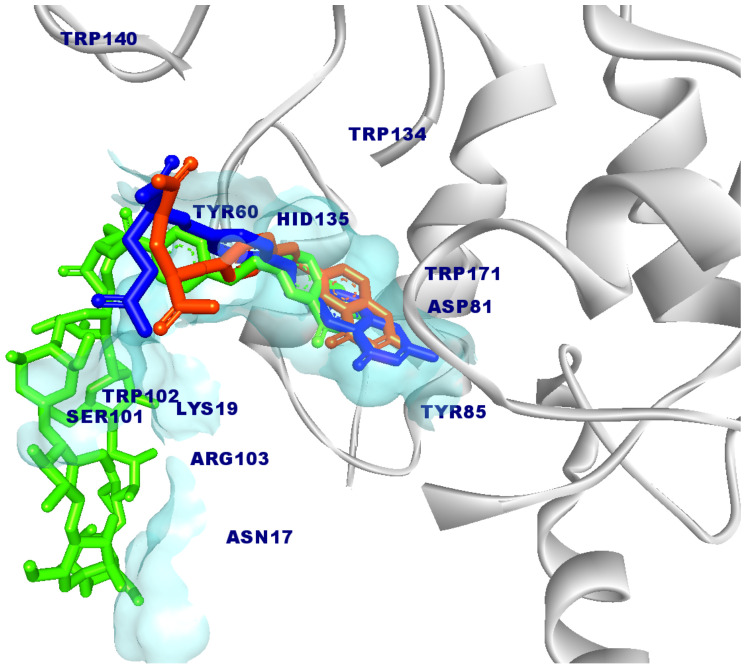
Superposition of docked models of FA (RMSD = 0.90 Å) and FA–βCD (RMSD = 5.04 Å), shown in orange and green colours, respectively, compared to the reference structure FA (blue) with FRα (PDB ID: 4LRH). The key amino acids that interact with the ligands are labelled in navy blue, and the binding site is colour-coded in transparent blue. Some regions of the protein are omitted to facilitate visualization.

Redocking of FA from the crystal structure was carried out first to validate the docking procedure. The FA molecule was extracted from the crystal structure of FA in complex with FRα (PDB ID:4LRH) [26] and redocked into the same binding site. Figure 2 shows that the docked pose of FA was similar to its crystallographic pose (RMSD = 0.90 Å), indicating that docking was able to reproduce the experimental result. Then, FA–βCD was also docked into the same binding site. Table 1 shows the docking scores (free binding energy, FEB) of the systems (FA– and FA–βCD) upon docking to FRα. Interestingly, FA–βCD (FEB = −15.20 kcal/mol) showed more negative free binding energy than FA (−13.20 kcal/mol), indicating a more favourable interaction between FA–βCD with FRα. Although the RMSD value of FA–βCD is 5.04 Å, its FA moiety was bound to the active site of FRα in a similar binding pose (Figure 2).

**Figure 3:**
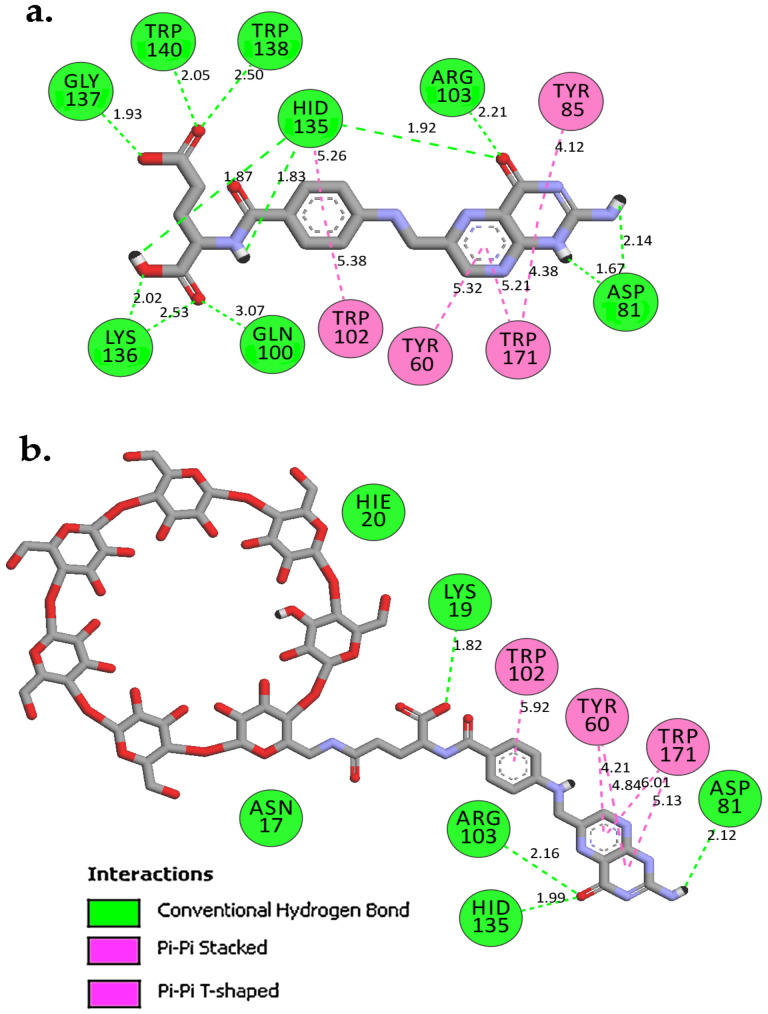
2D-Interaction analysis of docked models of (**a**) FA and (**b**) FA–βCD with FRα binding site.

As with the FA–FRα complex (Figure 2 and Figure 3a), the folate moiety of the FRα–FA–βCD complex was also stabilized by polar amino acids such as ASP81, TYR60, ARG103, HIS135, and nonpolar residues such as TRP102 and TRP171 [27]. The main interactions of these residues were with pteroate moiety (pteridine ring with PABA (p-amino benzoic acid)) (Figure 3b). These observations are similar to those in the crystal structure of human FRα complexed with FA where this pteroate moietywas buried inside the deep binding pocket of FRα [26]. It is noted that the βCD did not enter the binding site, and only formed an interaction with the amino acid at the surface of FRα such as LYS19. Interestingly, conjugation to βCD resulted in the FA to elude its H-bonds interaction between its glutamic acid moiety with LYS136, GLY137, TRP138, and TRP140, a behaviour observed in our previous study [27]. In addition, Figure 3b shows that the residue TYR85 lost its π–π stacking interaction with the pteridine ring from the FA structure of the FA–βCD. This could be because of the excluded volume induced by βCD. Presumably, this affects the position of surrounding residues in the binding site, and it may cause the FA moiety to drift a little from the binding site (Figure 2). This docked pose of FA–βCD was then used as the starting structure for the MD simulation to investigate further on the mechanism of binding of FA–βCD onto FRα.

### 2.2. Molecular Dynamics (MD) Simulation

#### 2.2.1. Stability of the Simulated Systems

**Figure 4:**
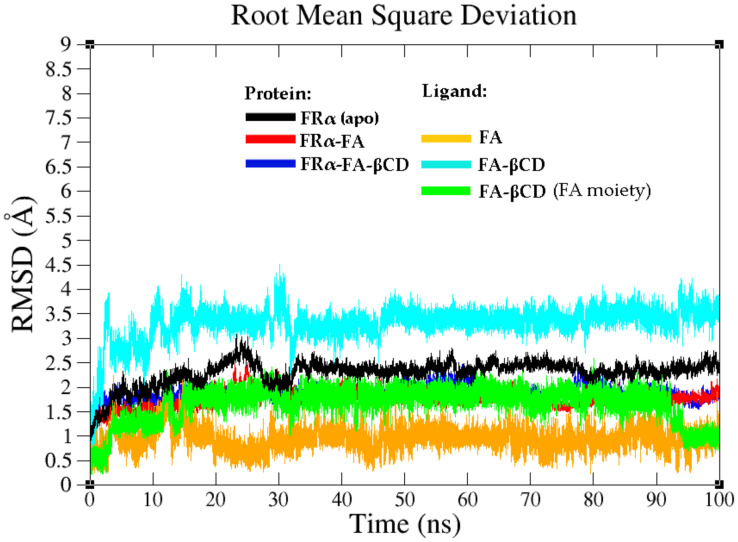
The root mean square deviation (RMSD) plots of the protein and ligand backbone atoms for the selected systems. Apo-FRα (black), FRα–FA (red and orange), and FRα–FA–βCD (blue, cyan, and green).

Understanding the atomic level interactions and the resulting structural characteristics is important for the targeted drug delivery application of βCD conjugated with FA. In order to explore the binding stability of the systems and gain a deeper understanding of the dynamical behaviour of the complexes, 100 ns MD simulations of FRα in complexed with FA and FA–βCD were performed. The behaviour of the complex systems was also compared with that of the ligand free system (apo-FRα). Prior to the simulation, apo-FRα was set up by removing the folic acid from the same crystal structure used as the starting structure of the FRα–FA system. The stability of the simulated systems was investigated by tracking the root-mean-square deviation (RMSD) of the protein backbone and ligand atoms during the 100 ns of MD simulations, as shown in Figure 4. In general, all systems were found to reach equilibrium after 35 ns with stable RMSD values ranging from 1.9–2.4 Å during the simulation time.

Figure 4 shows that the apo-FRα backbone has the highest RMSD compared to the holo or ligand-bound systems (FRα–FA and FRα–FA–βCD). This observation is not surprising as the apo-FRα was built from the FA–bound complex [26]. Removal of FA from the holo form (folate complex) might result in the transition from one biological trafficking state to another in the system, resulting in higher RMSF especially in regions where the ligands are bound [26,27], which in this case is the folate-binding pocket [26]. The FRα–FA–βCD system reached a stable and equilibrated dynamical state relatively quick, i.e., in less than 10 ns, and continued to fluctuate (protein backbone) within a stable conformational ensemble with an average RMSD value of 1.9 Å throughout the 100 ns simulations. The high RMSD showed by FA–βCD might be due to its βCD moiety as the FA moiety of FA–βCD (green) showed much lower RMSD values compared to the whole ligand (light blue).

**Figure 5:**
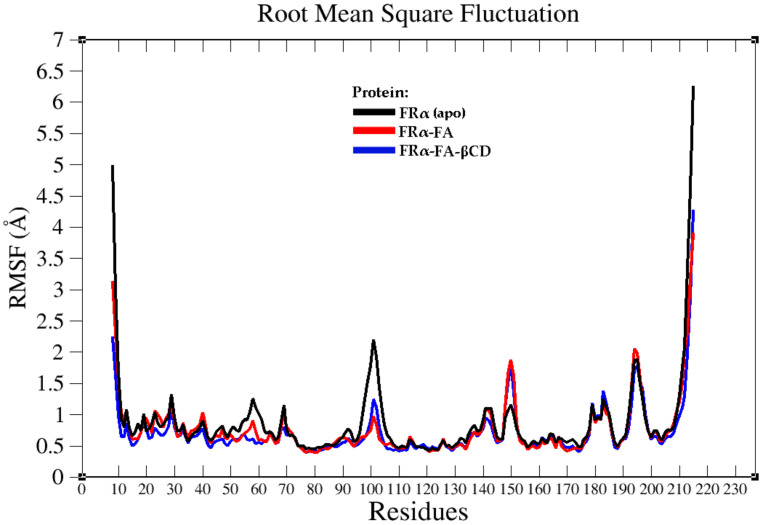
RMSF graph of the FRα backbone atoms for the three systems throughout the 100 ns MD simulation time.

In addition, the structural flexibility of the simulated systems was also investigated by measuring the root mean square fluctuation (RMSF) for FRα backbone atoms in all systems throughout the 100 ns MD simulation time, as illustrated in Figure 5. The average residual fluctuation values of the FRα backbone atoms (except for terminal residues) for all systems were less than 2.1 Å (Figure 5). A high degree of flexibility (particularly the residues 17–30, 38–58, 95–104, and 136–150), was expected due to the high percentage of unstructured segments inside the sequence [28].

**Figure 6:**
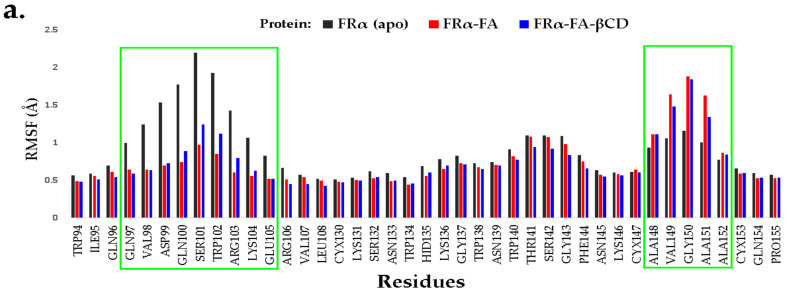

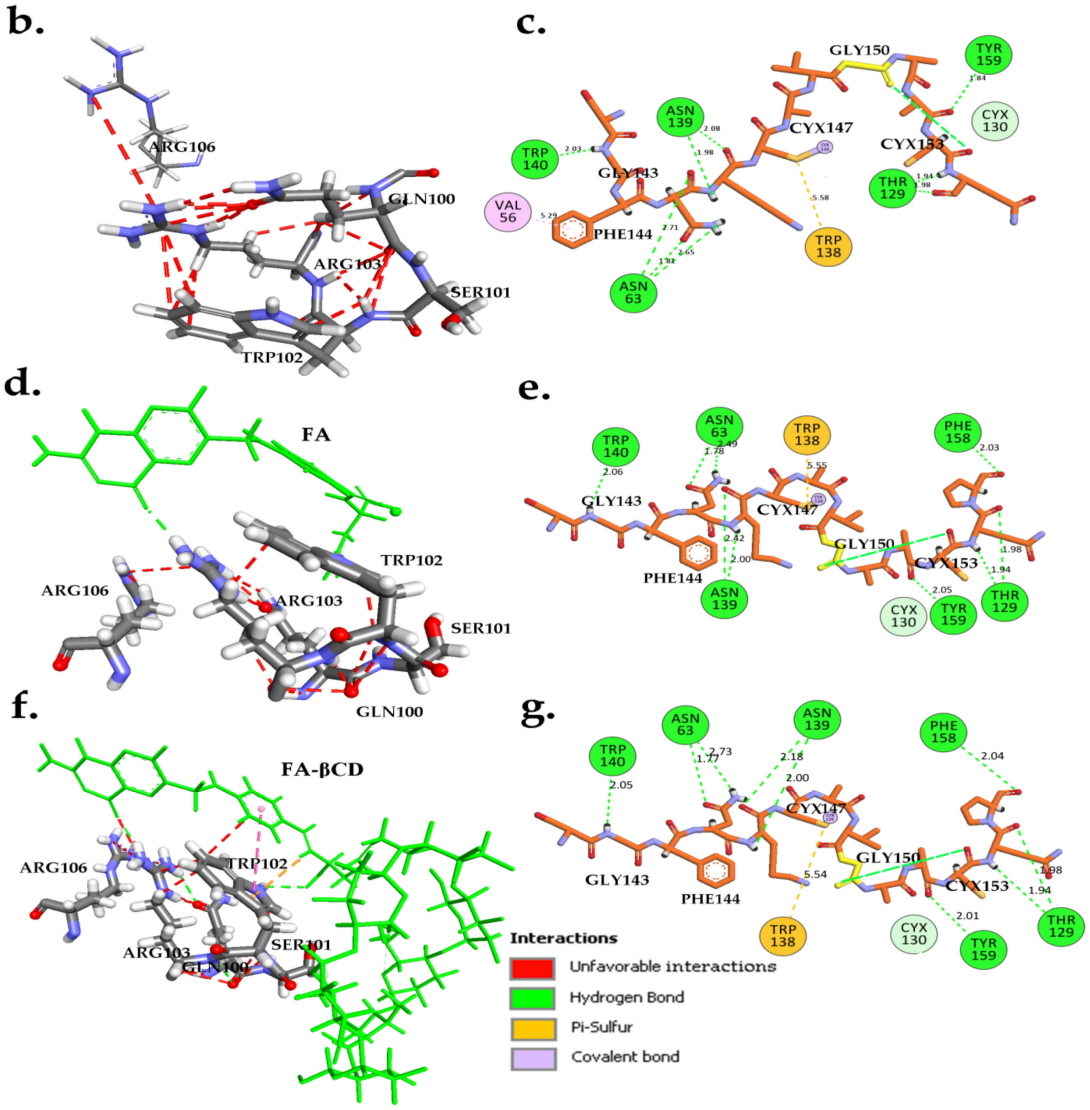
(**a**) RMSF table plot of the FRα backbone atoms for the three systems within the regions 94–108 and 130–155 throughout the 100 ns MD simulation time. The average structure (0–100 ns) for the most fluctuating residues in the systems apo-FRα (**b**,**c**), FRα–FA (**d**,**e**), and FRα–FA–βCD (**f**,**g**) were illustrated using Biovia Discovery Studio Visualizer.

An interesting observation was noted for the apo-FRα system, where high fluctuation occurred within the region of residues 97–105, with its peak at SER101 (Figure 6a). In the holo systems, SER101 was found to form strong H-bonds with the ligands (glutamate region) together with guanidium groups of ARG103 and ARG106 with the pteridine moiety [26]. Thus, it is expected that the loss of these interactions will affect the flexibility of these amino acid residues. Unfavourable interactions (charge repulsions) between the guanidium groups of ARG103 and ARG106 were also observed. The guanidium group of ARG103 also created a donor–donor clash with the amido group of GLN100 in the holo systems. These unfavourable interactions resulted in ARG103 to push the benzimidazole ring of TRP102 and compelled SER101 (located between GLN100 and TRP102) to fluctuate in order to reduce the unstable conditions (Figure 6d,f).

TRP102 is responsible for stabilizing the folic acid aminobenzoate through hydrophobic interaction as well as the glutamate group through hydrogen bond interaction [26]. As such, in the holo systems, this residue did not fluctuate much as it is involved in the binding of both FA and FA–βCD. Higher fluctuations were also observed for residues at the N- and C-termini in all systems which were due to the fact that these regions were not restrained [28]. Similar to that observed by Della-Longa et al., compared to apo-FRα, the presence of FA and FA–βCD in the holo systems reduced the mobility of most residues which are involved in both hydrophobic and electrostatic interactions with the ligands [28].

Figure 6c,e,g show the inter-residues interactions for residues 142–155, where an increase in RMSF around GLY150 was observed. All residues in this fragment made significant H-bond interactions with other residues surrounding them with the exception of residues 148–152. This possibly explains the fact that these residues exhibited higher RMSF compared to other residues in the fragment. Glycine residue is unique as it does not carry aside chainthus providing greater freedom for flexibility for the adjacent residues [29]. Thus, it is not surprising that the adjacent residues ALA148, VAL149, ALA151, and ALA152 in the holo systems also had higher RMSF than other adjacent residues in apo-FRα system (Figure 6a). When the RMSF values of the same fragment in apo-FRα (Figure 6b,c) were compared to that in the FRα–FA and FRα–FA–βCD systems, only a small significant difference in terms of its mobility (RMSF less than ~0.7 Å) was observed. The increase in the mobility of the GLY150 in the halo system might be due to the fact that PHE144 lost its interactions with the VAL56 (Figure 6e,g), which then led to the flipping of the phenyl ring and might affect the conformation of the surrounding amino acids. It is worth noting that the distances between the sulphur atoms of the cysteine residues in the region (130–155) were almost the same for all systems with an increase or decrease of 0.1 Å. This indicates that the disulphide bridges and the integrity of the receptor structure are still intact.

**Figure 7:**
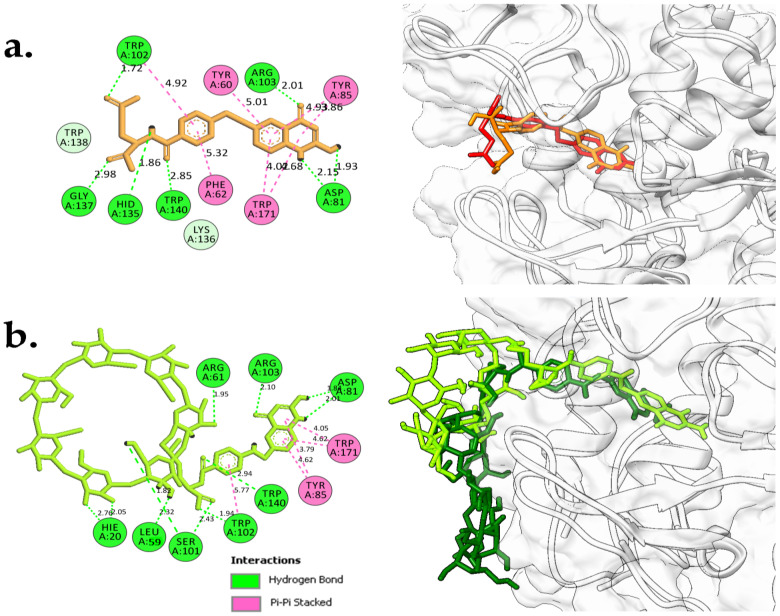
2D-Interaction analysis (models on the left) of average simulated structures (90–100 ns) for (**a**) FRα–FA (orange) and (**b**) FRα–FA–βCD (lime green), and superimposition with docked structures (models on the right) for FRα–FA (red) and FRα–FA–βCD (dark green). These models were generated using UCSF Chimera 1.13 and BIOVIA Discovery Studio Visualizer.

Superimposition of the average structure of the protein complexes from the stable region (90–100 ns) with the best docked pose (Figure 7) revealed that conjugation with βCD still allowed FA to maintain its binding with FRα, with the pteridine ring located within the FRα binding site, and the gamma carboxylate group from the glutamate portion at the entrance of the binding site as with that observed in the crystal structure (4LRH.PDB) [26]. ASP81 form two strong H-bonds with FA–βCD; one with a pteridine ring at N5 at a distance of 2.01Å and the other with N7 (1.84 Å) (Figure 7b). This observation is consistent with previous studies that showed that ASP81 interacted with the pteridine ring and is considered as a key contributor to the high folate affinity [26,27,30]. In FA–FRα (Figure 7a), two H-bonds were also observed between the pteridine ring with ASP81 (1.93 Å and 2.15 Å). However, in FRα–FA–βCD, the H-bond with HIS135 was lost, together with the H-bond formed by GLY137 with the glutamic acid moiety; but the GLY137–FA H-bond at this site was replaced by SER101–FA. Nonetheless, the H-bond formed between TRP140 and the PABA moiety was still preserved. In addition, TRP102 also formed π−π interaction with the phenyl ring of PABA. The π−π interactions were also formed between the pteridine ring and TYR85 and TRP171. Besides these H-bonds, conjugation with βCD had allowed additional H-bonds to be formed between the βCD moiety with SER101, ARG61, LEU59, and HIS20. This increased the number of H-bond interactions in the FA–FRα complex from 7 to 11 in the FRα–FA–βCD system. This suggests that βCD conjugation to FA did not adversely affect the stability of the ligand’s interaction with the FRα binding site. On the contrary, the conjugation with βCD improved the stability as seen from Figure 5, where the RMSF values for all the residues (except the residues 100–104 and 149–151) in FRα–FA–βCD are lower than in the FRα–FA system.

#### 2.2.2. Radius of Gyration Analyses

**Figure 8:**
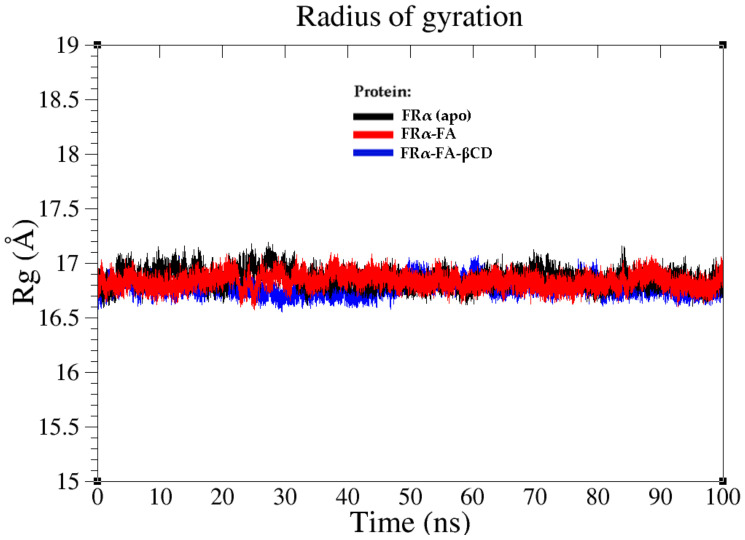
Radius of gyration (Rg) plots of the FRα backbone atoms of the three systems at MD interval time (0–100 ns); Apo-FRα (black), FRα–FA (red), and FRα–FA–βCD (blue).

Radius of gyration (Rg) is a parameter that determines the steady-state conformation of a total system and the macromolecule’s compactness [31]. Figure 8 illustrates that the three systems showed consistent Rg values of around 16.8 Å throughout the MD simulation time of 0–100 ns, with an exception for the apo-system, where a higher Rg value was observed from 0–35 ns. Similar to the RMSD observation, this is possibly due to the fact that the system was adjusting itself after the removal of FA in the initial crystal structure.

#### 2.2.3. Hydrogen Bond (H-Bond) Properties

**Figure 9:**
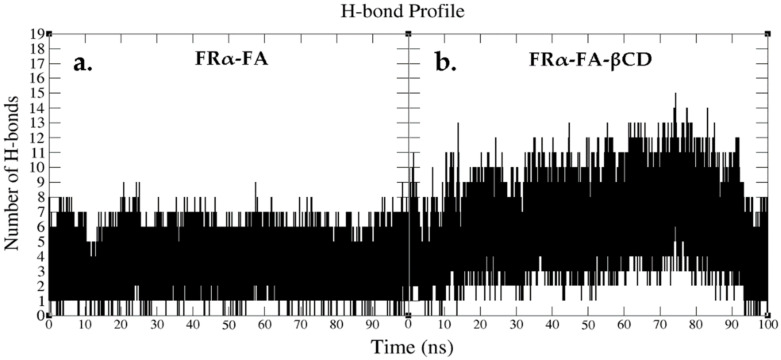
Hydrogen bond profile obtained from MD simulation (0–100 ns) for (**a**) FRα–FA and (**b**) FRα–FA–βCD.

H-bonds are essential for protein folding and protein–ligand interactions [32,33]. It is well known that ASP81 is the key residue in the FRα binding site, playing a critical role in increasing ligand binding affinity and anchoring the FA pteridine region deep within the site [27,30,34]. Specifically, the crystal structure used in this study demonstrated that ASP81 formed strong H-bonds with N1 and N2 atoms in the pteridine ring of FA. The pteridine ring also formed two H-bonds with the arginine residues, ARG103 and ARG106, as well as with SER174 and HIS135 [26,28]. Hence, the current study was performed to explore the effect of the conjugated FA–βCDs on their mechanism of binding with FRα throughout the MD simulation time (100 ns), by analyzing the number of H-bonds created with the protein (Figure 9). Moreover, the H-bond occupancy of the conjugated systems was also analyzed throughout the MD simulation time (0–100 ns) and is presented in Table 2.

**Figure 10:**
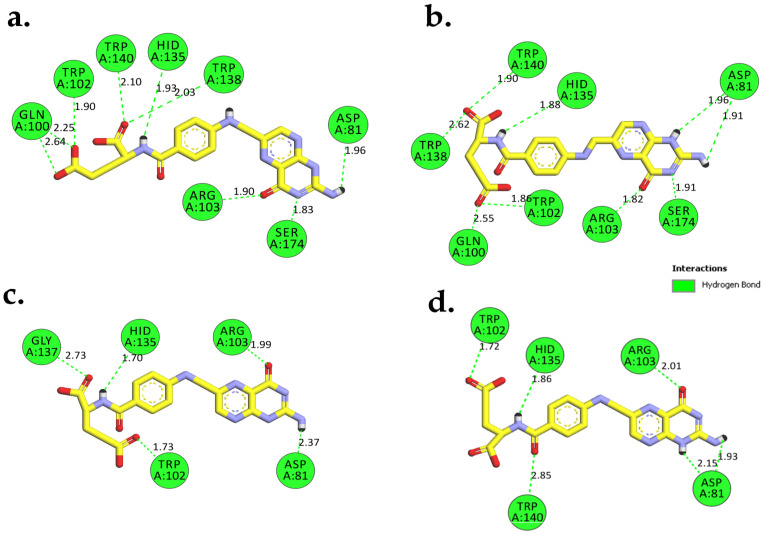
Models (2D-interactions) illustrate that the H-bonds of FA interacted with amino acids in the FRα binding site; (**a**) at 20.8 ns which demonstrated the optimum H-bond interactions (9 H-bonds), (**b**) 98.3 ns, (**c**) the average H-bonds (5 H-bonds) at 71.2 ns, and (**d**) 100 ns (6 H-bonds). These models were generated using Biovia Discovery Studio Visualizer. FA is shown as sticks (yellow C, red O, and blue N), and the residues of FRα are shown in green colour.

**Figure 11:**
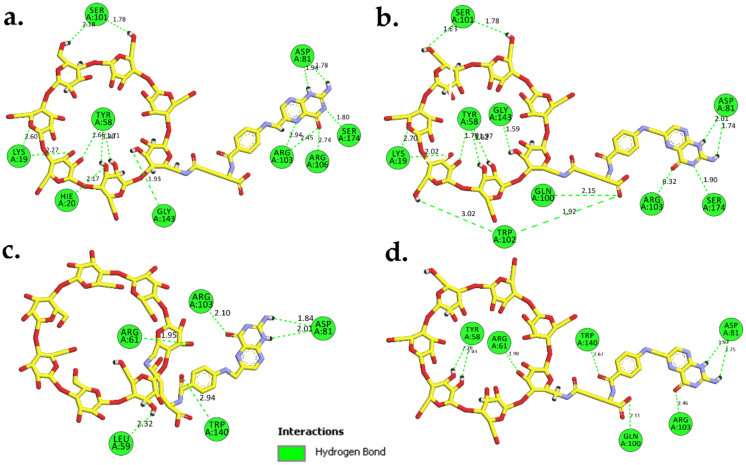
Models (2D-interactions) illustrating the H-bond interactions between FA–βCD and amino acids in the FRα binding site; optimum H-bond interactions (15 H-bonds) at (**a**) 74.4 ns and (**b**) 74.5 ns, (**c**) the average H-bonds (6 H-bonds) at 95.8 ns, and (**d**) at 100 ns (8 H-bonds). These models were generated using Biovia Discovery Studio Visualizer. FA–βCD is shown as sticks (yellow C, red O, and blue N), and the residues of FRα are shown in green colour.

In the FRα–FA system, the H-bond profile revealed consistent interactions throughout the MD simulation time with an average of five bonds (Figure 9a and Figure 10). In contrast to the FRα–FA system, the H-bond profile of FRα–FA–βCD (Figure 9b) showed ten H-bonds in the initial stage which fluctuated until 90 ns. At 74.4 ns and 74.5 ns, the system produced the best H-bond interactions (15 H-bonds) (Figure 11a,b). Subsequently, the H-bond profile abruptly decreased to an average of six bonds until 100 ns (Figure 9b and Figure 11c). It is worth noting (Figure 11a,b) that, except with HIS135, the folate moiety created six H-bonds with the amino acid residues, i.e., ASP81, ARG103, ARG106, and SER174, as observed in the crystal structure [27] and in our previous MD simulation [26].

It is interesting to note that βCD participated in forming nine H-bonds with the surrounding amino acids at the entrance of the binding site (Figure 11a,b). This could be due to the highly free rotation at the FA/βCD NH-CH_2_ junction and the massive network of hydroxyl groups present in βCD, which contributed to the ability to form high number of H-bonds with the surrounding amino acids. Consequently, in a βCD loaded system, in particular where host–guest interactions involved hydrophobic drug, the presence of many H-bond interactions between βCD and the protein target could aid in expanding the torus-shape of βCD, thus affecting the stability of the loaded system and leading to the release of the loaded hydrophobic drug outside the cell [35,36]. However, during the last MD simulation time (90–100 ns), the βCD’s capacity to form H-bonds had decreased on average from nine to two (Figure 11c), indicating the capability of the βCD loaded system to contain the drug upon binding to the receptor.

Table 2 shows the average H-bond occupancy, distance, and angles for the conjugated systems (FA and FA–βCD) in their interactions with key amino acids in the binding site throughout the MD simulation time (0–100 ns). In this analysis, hydrogen bonds were classified as strong (more than 60% occupied), moderate (30–60% occupied), and weak (10–30% occupied) based on their percentage of occupancy throughout the specific region of MD simulation [27,37,38]. The results showed that FA and FA–βCD bound to the FRα active site via H-bonds with varying tendencies, and ASP81 remaining as the key amino acid in the H-bond interaction. Interestingly, FA–βCD formed more H-bonds compared to FA during the MD simulation time (0–100 ns). The findings from FA–FRα system revealed the existence of two strong H-bonds between the OD1 and OD2 of ASP81 and the hydrogen atoms (H1 and H2) at the N1 and NE2 of FA’s pteridine ring, with 71.57% and 61.60% occupied during the 100 ns simulation, respectively, and an average distance of 2.81 Å and 2.81 Å, and an angle of 158.54° and 163.32°, respectively. Furthermore, there are two moderate H-bonds between HIS135 and ARG103 residues with hydrogen atom (H12) at N and O4 of the FA structure with occupancies of 57.68% and 37.07%, respectively.

The findings also showed that the FRα–FA–βCD system exhibited only one strong H-bond between OD1 of ASP81 and the hydrogen atom (H93) at the N7 of the pteridine ring of FA, with 62.16% occupancy, and with an average distance and angle of 2.81 Å and 163.10°, respectively. Furthermore, five moderately strong H-bonds were formed with ASP81 (OD2), SER174 (HG at OG), TYR58 (O), ARG103 (HH12 at NH1), and ASP (OD1) with an average occupancy of 54.81%, 46.72%, 46.64%, 44.15%, and 30.83%, respectively. The rest of the H-bond interactions, on the other hand, were relatively weak (Table 2).

#### 2.2.4. Binding Free Energy (MM-PBSA)

In this study, the Molecular Mechanics-Poisson Boltzmann surface area (MM-PBSA) program implemented in AMBER 18 [39,40] was used to calculate the free binding energies of FA and FA–βCD with FRα, with a neglected entropic contribution. Table 3 shows the calculated free binding energies $\left(\Delta G_{bind^{*}}\right)$ computed using the Molecular Mechanics-Poisson Boltzmann surface area (MM-PBSA) method implemented in AMBER 18 [41]. The predicted MM-PBSA energy of FA and FA–βCD toward FRα were −57.17 and −74.25 − kcal/mol, respectively. The corresponding energetic values revealed that the electrostatic term is the main contributor to the binding energies, in addition to the van der Waals term. However, the solvation energies, particularly the polar solvation energy, produced an unfavourable binding contribution. The MMPBSA results also show that the conjugation with βCD improved the binding affinity of FA towards FRα.

FA–βCD showed more favourable $\Delta G_{bind^{*}}$, with the highest contribution from the electrostatic and van der Waals terms, compared to that of FA (Table 3). This system was also more stable, as demonstrated by the RMSD of the protein backbone (Figure 4). The conjugated ligand, however, had higher RMSD values compared to unconjugated ligand.

**Figure 12:**
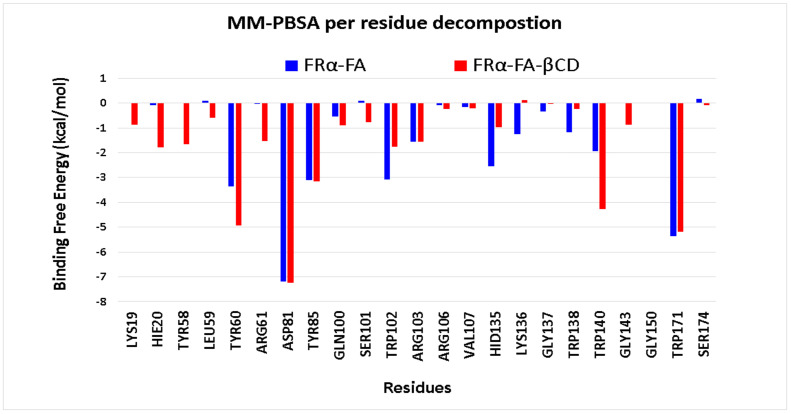
Individual amino acid binding free energy plots that significantly contribute to the interactions into the FRα pocket with the ligands (FA- and FA-βCD) in the last 50 frames of the MD trajectories. The X-axis represents the ID of the residue, and the Y-axis represents the binding free energy in kcal/mol.

The partitioning of free energy into additive contributions originating from different groups of atoms or force field terms has the potential to provide a relationship between the structure and biological activity of molecules [42]. This free energy contribution was further decomposed into the sum of free energies originating from the interactions of the protein with itself, its substituents, water, and ions [43]. Figure 12 illustrates the MM-PBSA per residue decomposition values for FRα residues in the holo systems (FRα–FA and FRα -FA–βCD). The analysis was carried out on the protein binding site residues [26,27], and on the highest mobile residues throughout the RMSF analysis. The plot revealed that ASP81 had the most negative energetic values (high interactions with the surrounding residues) in both the FRα–FA and FRα–FA–βCD systems with relatively the same contribution (~−7.10 kcal/mol), followed by TRP171. It was noted that residues LYS19, TYR58, LEU59, TYR60, ARG61, TRP140, and GLY143 demonstrated more favourable energy contributions in the FRα–FA–βCD system than those in the FRα–FA system, and they were also less mobile (dynamically stable), as shown by the RMSF values (Figure 5). This indicates that these residues may have a direct role in increasing the stability of the conjugated system.

## 3. Materials and Methods

### 3.1. Molecular System Setup

#### 3.1.1. Protein Preparation

The human FRα crystal structure (PDB ID: 4LRH) was downloaded from the Protein Data Bank database [26]. All water molecules and heteroatoms were eliminated using Biovia Discovery Studio Visualizer (San Diego, CA, USA, 2019) [44]. In order to prepare the molecular system, the PDB2PQR web service (https://pdb2pqr.poissonboltzmann.org/pdb2pqr, accessed on 14 April 2021) was utilized for additional calculations to the protein such as reconstructing missing atoms, adding hydrogens, and assigning atomic charges and radii with the SWANSON force field (AMBER ff99 charges with optimised radii) [45]. The protein was subjected to the most commonly used empirical pKa predictor (PROPKA3) to assign the protonation states for the ionizable groups, set at pH 7.00 [46]. Finally, the protein was uploaded to the MolProbity web service (http://molprobity.biochem.duke.edu/, accessed on 14 April 2021) to correct bad contacts, add hydrogen atoms, and flip HIS, GLU, and ASN residues [47].

#### 3.1.2. Ligand Preparation

In this study, FA was taken from the crystal structure [26] and conjugation to beta-cyclodextrin (βCD) (PubChem ID: 444041) was drawn at the gamma carboxylate group of the glutamic acid residue [24,48] using PerkinElmer ChemDraw 17.1 (PerkinElmer, Waltham, MA, USA) (Figure 1) [27]. Then, the ligands were subjected to energy minimization using Molecular Mechanics 2 (MM2) force field by PerkinElmer Chem3D 17.1 (PerkinElmer, MA, USA) [27].

#### 3.1.3. Molecular Docking

AutoDock Tool (The Scripps Research Institute, La Jolla, CA, USA) was used to add polar hydrogens and Kollman charges to the protein while Gasteiger charges were assigned to the ligands [49]. The rotatable bonds for the ligands were decreased to 8 based on the essential rotatable bonds in the FA scaffold. AutoDock Vina (The Scripps Research Institute, CA, USA) was used to simulate the docking process [50], where the grid box coordinate was set at center x = 44.532, y = 41.058 and z = 69.243 [27]. The size of the grid box was 40 × 40 × 40 (x, y, and z) with a spacing of 0.375Å, number of conformations = 20, exhaustiveness of the global search = 4, and maximum energy difference = 3. Biovia Discovery Studio Visualizer [44] and UCSF Chimera 1.13 (University of California San Francisco, CA, USA) [51] were used to visualize the 3D molecular interactions between the ligands and the protein.

### 3.2. Molecular Dynamics and Mechanics Simulations

The best-docked pose of the FRα–FA–βCD system was used as the starting structure to run the 100 ns MD simulation, while the apo-FRα system was created by removing FA from the crystal structure leaving FRα alone without the ligand to be simulated. MD simulations were performed using AMBER 18 (University of California San Francisco, CA, USA) [27,41]. The AMBER ff14SB force field and the general AMBER force field (GAFF) were applied on FRα and the ligands, respectively. All ligands were subjected to AM1-BCC model charges using the ANTECHAMBER tool in the AMBER suite. Apo-FRα system was developed from the same human FRα crystal structure. Each system was solvated by dipping it in an octahedral box of TIP3P water, where distance between the protein edge and box was 10 Å, and the system was neutralized by adding four counter ions (chlorine Cl^−^). After solvation and neutralization, the followings were recorded for each system: FRα–FA–βCD system consisted of 9327 atoms while apo-FRα (ligand free) system consisted of 8980 atoms.

The simulation protocol consisted of three minimization steps, whereby the first step includes 5000 cycles of conjugate gradient, 2000 for the second step, and 1000 for the third step, with periodic boundary conditions at constant volume to eliminate the collision contacts between the macromolecule and the solvent, and to relax the system. The system was then gradually heated in three steps from 0–310 K for 1 ns on all backbone atoms in each step using the Langevin dynamics thermostat with a coupling time of 0.2 ps. During the heating process, the NVT ensemble was used. Next, the equilibration of the protein atoms and the surrounding solvent was performed in three steps for 2 ns each, and the SHAKE algorithm [33] was also utilized to constrain all bonds involving hydrogen.

MD simulation was carried out for 100 ns. Trajectory analysis was done using CPPTRAJ to inspect the Root Mean Square Deviation and Fluctuation (RMSD and RMSF) values, radius of gyration (Rg), and hydrogen bond (H-bond) that were involved in the interaction between each ligand and the protein. QtGrace 0.2.6 (Boston, MA, USA) was used to create the graphs.

### 3.3. Free Binding Energy Calculation by MM-PBSA

The free binding energy (FEB) of the different complex systems was calculated using the Molecular Mechanics-Poisson Boltzmann Surface Area (MM-PBSA) method in the AMBER 18 program (University of California San Francisco, CA, USA). MM-PBSA method combines the molecular mechanics and continuum solvent models, and the Gibbs free binding energy (ΔG) calculated using the MM-PBSA method [40]. All energetic analyses were done using a single trajectory approach, where snapshots were taken for each of the protein–ligand complexes, protein, and ligands of the performed MD simulations. Energy calculation was performed for every 10 ps (total 1000 snapshots) from the last 10 ns of the trajectory (90–100 ns) using the MM-PBSA.py module of AMBER 18, with a salt concentration of 0.150 M, and without quasi-harmonic entropy approximation, in order to obtain a close approximation to true molecular volume, albeit in an average sense [27]. 

In addition, free energy decomposition for each complex system was examined to obtain information on the important binding site residues involved in ligand binding. The energy contribution of each residue (per residue decomposition) was divided into three parts: van der Waals energy (∆G_vdw_), intermolecular electrostatic energy (∆G_ele_), and solvation energy (∆G_sol_) due to solvent effect, which was a sum of the polar solvation energy (∆G_PB_) and the non-polar solvation energy (∆G_SA_) [41,42,43].

ΔG_residue_pair_ = ΔG_vdw_ + ΔG_ele_ + ΔG_solvation_ = ΔG_vdw_ + ΔG_ele_ + ΔG_PB_ + ΔG_SA_ (1)

where ΔG_vdw_ and ΔG_ele_ are nonbonded van der Waals and electrostatic interactions between two residues, respectively [41]. As a result, the combination of those energetic components may have a correlation with experimental binding affinity values [39]. The per-residue energy was calculated using the MM-PBSA.py implemented in the AMBER 18 package to calculate the per-residue decomposition for the last 50 frames of the trajectory.

## 4. Conclusions

In this study, the systems apo-FRα, and two holo systems of FRα bound with FA and FA conjugated beta-cyclodextrins (FA–βCD) were successfully simulated. Our findings suggest that FA–βCD is more dynamically stable than FA. The docking results showed that all ligands entered into the binding site of FRα (docking score < −15 kcal/mol) and relatively bound with the same binding pose. Molecular dynamic simulation showed that the binding of FA and FA–βCD on FRα did not affect protein stability. FA–βCD had more consistent interactions and more favourable individual residue binding energies than the FA. The holo systems’ residues HIS20, TRP102, HIS135, TRP138, TRP140, and TRP171 were revealed to have more favourable energy contributions and were less mobile than those in the apo-FR system (dynamically stable). This shows that these residues may play a direct role in the system stability. H-bond analysis and per residue free energy decomposition analysis support the previous finding of ASP81 as the key residue that influence the binding of ligands into the binding site of FRα. This work serves to provide an understanding on the effect of conjugation of βCD to the activity and stability of folic acid to FRα. This is important in the understanding of the targeted mechanism of folic acid and the folic acid conjugated drug delivery system in the treatment of cancer, thus this can be the basis for future studies on the inclusion complex or drug loading studies.

## Figures and Tables

**Table 1:** AutoDock Vina docking scores of FA and FA–βCD docked against FRα.

Ligand	Free Energy of Binding, FEB (kcal/mol)	RMSD *
FA	−13.20	0.90 Å
FA–βCD	−15.20	5.04 Å

* The calculated RMSD values were referred to as deviation of the FA structure from the crystal structure of FA in 4LRH.PDB [26]. * RMSD calculated only on the FA moiety of the molecule.

**Table 2:** H-bond occupancies for the complexed systems FRα–FA and FRα–FA–βCD in the MD simulations (0–100 ns).

System	H-Bond Acceptor (Atom ≅ Res)	H-Bond Donor (Atom ≅ H)	Donor (Atom ≅ Res)	H-Bond Occupancy (%)	Average Distance (Å)	Average Angle
FA	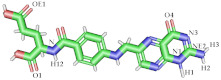					
	ASP81 ≅ OD1	ASP81 ≅ OD1	ASP81 ≅ OD1	ASP81 ≅ OD1	ASP81 ≅ OD1	ASP81 ≅ OD1
	ASP81 ≅ OD2	ASP81 ≅ OD2	ASP81 ≅ OD2	ASP81 ≅ OD2	ASP81 ≅ OD2	ASP81 ≅ OD2
	HIS135 ≅ O	HIS135 ≅ O	HIS135 ≅ O	HIS135 ≅ O	HIS135 ≅ O	HIS135 ≅ O
	FA ≅ O4	FA ≅ O4	FA ≅ O4	FA ≅ O4	FA ≅ O4	FA ≅ O4
	ASP81 ≅ OD2	ASP81 ≅ OD2	ASP81 ≅ OD2	ASP81 ≅ OD2	ASP81 ≅ OD2	ASP81 ≅ OD2
	FA ≅ O1	FA ≅ O1	FA ≅ O1	FA ≅ O1	FA ≅ O1	FA ≅ O1
	FA ≅ OE1	FA ≅ OE1	FA ≅ OE1	FA ≅ OE1	FA ≅ OE1	FA ≅ OE1
	FA ≅ N3	FA ≅ N3	FA ≅ N3	FA ≅ N3	FA ≅ N3	FA ≅ N3
	ASP81 ≅ OD2	ASP81 ≅ OD2	ASP81 ≅ OD2	ASP81 ≅ OD2	ASP81 ≅ OD2	ASP81 ≅ OD2
FA–βCD	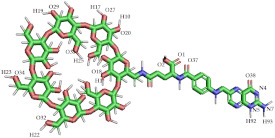					
	ASP81 ≅ OD1	FA ≅ H93	FA ≅ N7	62.16	2.81	163.10
	ASP81 ≅ OD2	FA ≅ H92	FA ≅ N5	54.81	2.81	159.98
	FA ≅ N4	SER174 ≅ HG	SER174 ≅ OG	46.72	2.85	160.9
	TYR58 ≅ O	FA ≅ H10	FA ≅ O20	46.64	2.74	160.27
	FA ≅ O38	ARG103 ≅ HH12	ARG103 ≅ NH1	44.15	2.85	151.81
	ASP81 ≅ OD1	FA ≅ H92	FA ≅ N5	30.83	2.82	159.09
	ASP81 ≅ OD2	FA ≅ H93	FA ≅ N7	28.96	2.82	162.8
	TYR58 ≅ O	FA ≅ H17	FA ≅ O27	27.29	2.71	161.37
	SER101 ≅ O	FA ≅ H24	FA ≅ O34	21.77	2.79	154.15
	FA ≅ O1	TRP102 ≅ HE1	TRP102 ≅ NE1	21.43	2.85	153.57
	FA ≅ O37	TRP140 ≅ HE1	TRP140 ≅ NE1	17.82	2.86	148.2
	SER101 ≅ OG	FA ≅ H22	FA ≅ O32	16.09	2.79	157.44
	FA ≅ O29	TYR58 ≅ HH	TYR58 ≅ OH	15.6	2.79	161.48
	SER101 ≅ O	FA ≅ H25	FA ≅ O35	15.37	2.72	159.55
	GLY143 ≅ O	FA ≅ H1	FA ≅ O18	14.29	2.76	163.16
	FA ≅ O38	SER174 ≅ HG	SER174 ≅ OG	11.27	2.86	148.02
	FA ≅ O20	HIS20 ≅ HE2	HIS20 ≅ NE2	11.23	2.88	152.09
	FA ≅ O18	ARG61 ≅ HH11	ARG61 ≅ NH1	10.43	2.88	158.11

**Table 3:** Binding free energies (MM-PBSA) for FRα–FA and FRα–FA–βCD from MD simulation trajectories (90–10 ns). Molecular docking values from AutoDock (ADT) Vina for the complexes are also included in the table.

Complex with FRα	$\Delta G_{bind^{*}}$ kcal/mol	VDW kcal/mol	EEL kcal/mol	G_polar_ kcal/mol	G_non-polar_ kcal/mol	ADT Vina kcal/mol
FA	−57.17 ± 0.12	−56.14 ± 0.10	−89.17 ± 0.26	94.61 ± 019	−6.47 ± 0.01	−13.20
FA–βCD	−74.25 ± 0.26	−92.71 ± 0.16	−119.47 ± 0.63	148.87 ± 0.48	−10.95 ± 0.01	−15.20

$\Delta G_{bind^{*}}$: binding free energy, VDW: van der Waals, EEL: electrostatic, G_polar_: polar solvation energy, G_non-polar_: non-polar solvation energy.

## Data Availability

Not applicable.
